# Endourologic Treatment for Aggressive Angiomyxoma of the Bladder

**DOI:** 10.1089/cren.2018.0106

**Published:** 2019-03-18

**Authors:** Rogério Cordeiro Filho, Aline A. Carvalho, Renan A. Carvalho, Manuela P. Cordeiro, Gabriel S. Cordeiro, Carlos D. Teixeira, Rogério S. Cordeiro, Renato N. Pedro

**Affiliations:** ^1^ Faculdade São Leopoldo Mandic, Campinas, Brazil.; ^2^ Uromed Day Hospital, Juazeiro, Brazil.; ^3^ Laboratório Bacchi, Botucatu, Brazil.

**Keywords:** aggressive angiomyxoma, endourologic, bladder tumor, transurethral resection of bladder tumor, TURBT

## Abstract

***Background:*** Aggressive angiomyxoma (AA) is a rare tumor that usually appears in the female pelvic and perineal regions. It commonly has infiltrative behavior and high local recurrence risk. We report an unusual presentation of AA, originating in a female patient's bladder.

***Case Report:*** A 43-year-old female patient presented with recurrent urinary tract infection for 6 months; ultrasonography showed a bladder tumor that was diagnosed as AA by immunohistochemistry and treated with complete transurethral resection.

***Conclusion:*** Transurethral resection can be an effective approach for the treatment of AA.

## Introduction

Aggressive angiomyxoma (AA) is an uncommon mesenchymal tumor that usually affects adults, commonly arising in the soft tissues of pelvic and perineal regions. Approximately 350 cases of the disease have been reported in literature, with a female to male ratio of 6.6:1. Considering the absence of typical signs and symptoms, diagnosis is frequently made by immunohistochemistry after surgical excision.

The standard treatment is the complete resection of the tumor, followed by long-term radiologic monitoring because of the high recurrence rate.

## Case Report

An otherwise healthy 43-year-old woman, presented with dysuria, increased urinary frequency, and recurrent urinary tract infection for the past 6 months. Physical examination revealed no abnormalities.

**FIG. 1.**
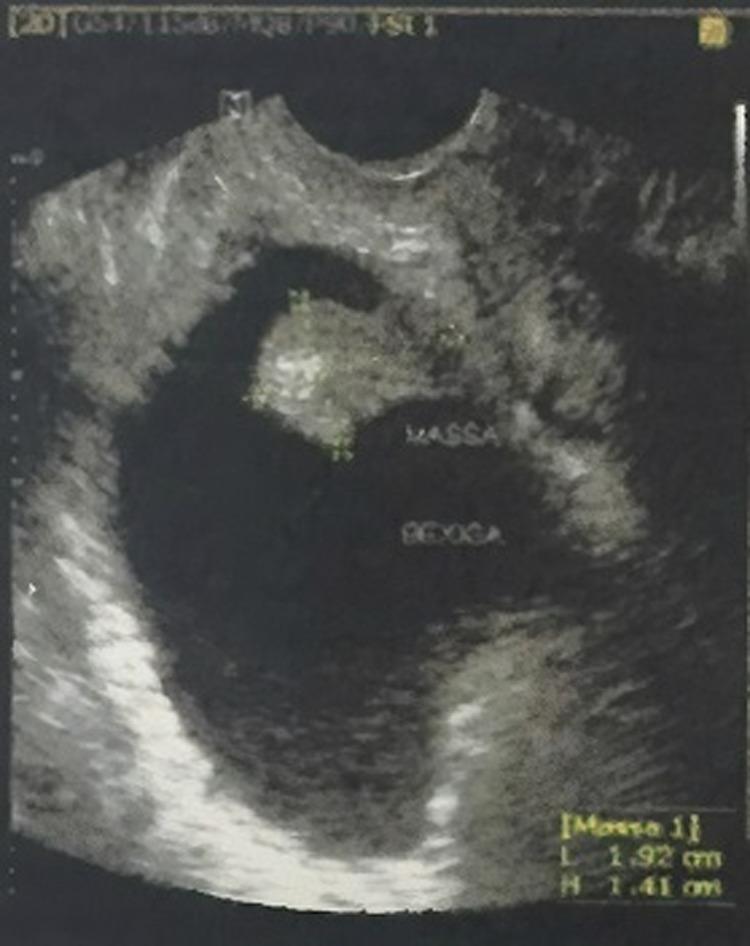
Transvaginal pelvic ultrasonography revealing hyperechogenic nodule.

Image work-up was indicated and transvaginal pelvic ultrasonography showed a pedicled bladder nodule, vascularized, hyperechogenic, with 1.9 × 1.4 × 1.8 cm of diameter. The patient was then admitted for an endoscopic evaluation and treatment of the exophytic intravesical mass (Fig. 1).

**FIG. 2.**
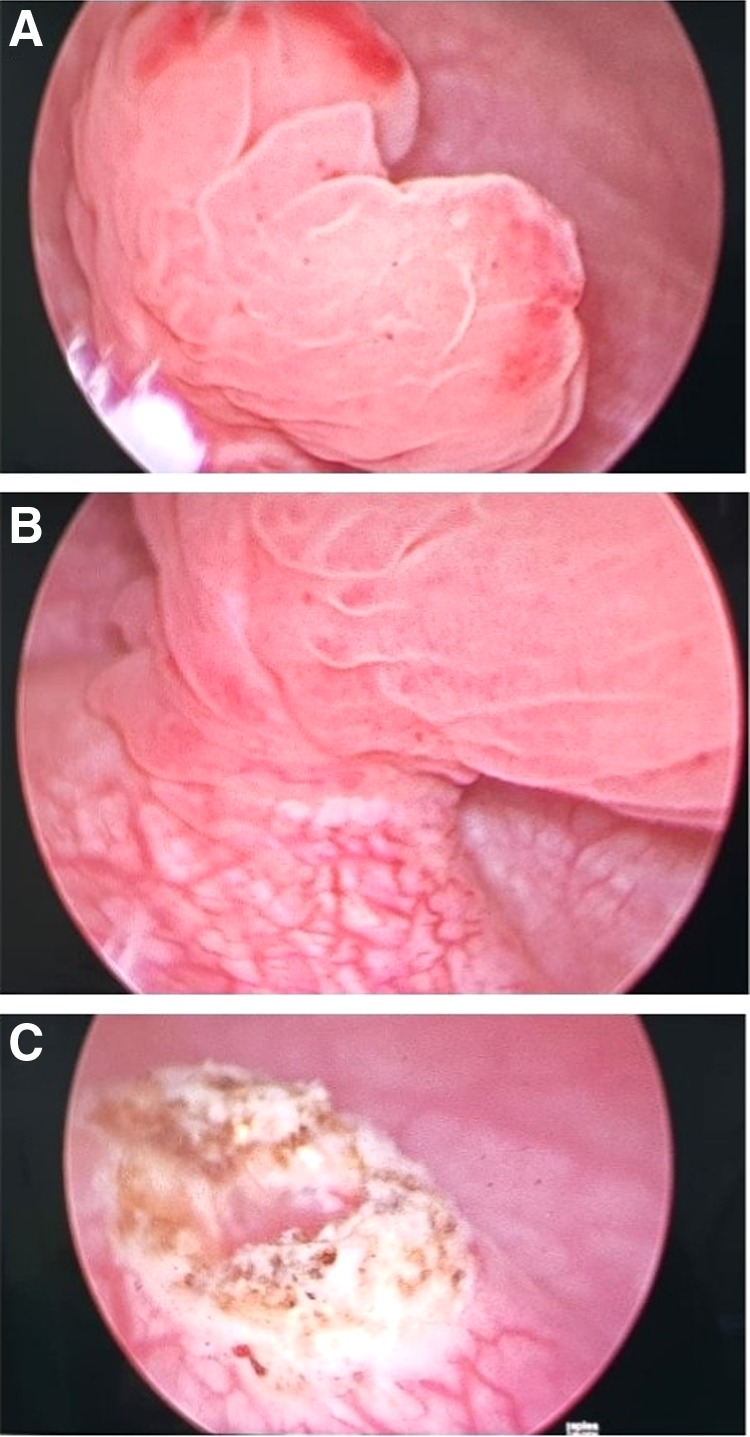
**(A, B)** Cystourethroscopy showing pedicled mass arising from the right ureteral orifice. **(C)** Postresection area showing no signs of remnant tumor.

**FIG. 3.**
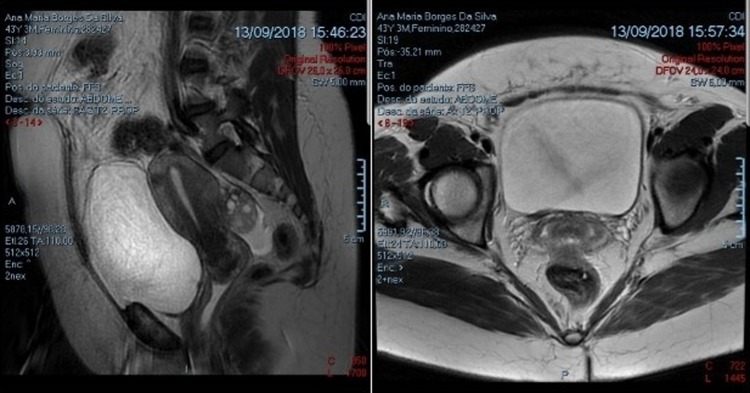
Follow-up magnetic resonance imaging 2 months after procedure showing no abnormalities.

Cystourethroscopic examination diagnosed a pedicled reddish mass projecting from the top of the right ureteral orifice with 6 cm diameter (Fig. 2). Complete standard transurethral resection with spinal anesthesia was performed. The exophytic mass was resected with an incision on the pedicle, after cautious withdrawal of the lesion through the urethra (Fig. 2). The procedure was uneventful and the patient was discharged from the hospital on day 1 post-TURBT. Follow-up was done by magnetic resonance imaging (MRI) 2 months after procedure, revealing no signs of abnormalities (Fig. 3).

**FIG. 4.**
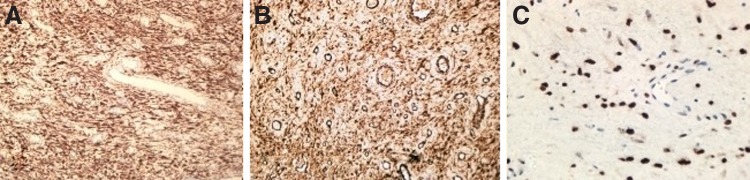
Immunoreactivity was shown for desmin **(A)**, muscle-specific actin **(B)**, and estrogen receptors **(C)**.

Histopathologic examinations featured dilated glands, chronic inflammatory infiltrate, edematous, vascularized, and loose stroma covered in transitional epithelial tissue without atypias. Immunohistochemistry study has shown spindled and stellate cell proliferation scattered in both myxoid and collagenic stroma with numerous blood vessels of varying caliber. Immunoreactivity was shown for desmin, muscle-specific actin, and estrogen receptors compatible with AA (Fig. 4).

Surveillance with cystourethroscopy every 3 months and annual MRI are planned because of the high recurrence potential of the AA.

## Discussion

The AA is a rare myxoid neoplasm, first described by Steeper and Rosai, in 1983, often affects soft tissues in perineal and pelvic zones of women in reproductive age.^1^ Some cases were described in men, commonly striking scrotal and inguinal areas. It usually presents infiltrative nature, low growth, and high recurrence rates, emphasizing its aggressive character.^2^ Clinically, it can be asymptomatic or have nonspecific symptoms; therefore, diagnosis is usually challenging.

Ultrasonography often presents hypoechogenic soft tissue mass with cystic feature. CT-scan images are variable, although it can present a well-defined homogenous mass. MRI commonly presents an isointense mass on T-1 weighted images and hyperintense on T-2 weighted images.^3^

On macroscopic analysis, the tumor may present hemorrhagic areas, exhibiting both reddish and grayish colors. It has infiltrative character, revealing irregular borders with a soft surface area. Histologic analysis may show the presence of blood vessels with varying caliber on connective tissue. Small eosinophilic stellate cells can be seen scattered in the stroma, without atypias.^1,2^

Immunohistochemistry study might exhibit immunoreactivity to different combinations of desmin, smooth muscle actin, muscle-specific actin, vimentin, estrogen and progesterone, CD34 receptors.^4^

The gold standard treatment is the complete resection of the tumor, although incomplete resection is commonly performed because of the high infiltrative behavior. Long-term surveillance with radiologic imagery is advisable on account of the high recurrence rates.

This case report shows an atypical AA presentation that was treated by a minimally invasive endourologic approach. Symptoms mimicked common lower urinary tract symptoms refractory to medical treatment, therefore emphasizing the importance of adhering to treatment guidelines for lower urinary tract symptoms and radiologic investigation of abnormal cases.

## Conclusion

Despite the highly infiltrative behavior of the AA, transurethral resection can be an effective approach for treatment when dealing with a primary bladder mass presentation.

## Abbreviations Used

AA: aggressive angiomyxoma
CT: computed tomography
MRI: magnetic resonance imaging
